# SMYD family in cancer: epigenetic regulation and molecular mechanisms of cancer proliferation, metastasis, and drug resistance

**DOI:** 10.1038/s12276-024-01326-8

**Published:** 2024-11-01

**Authors:** Tae-Su Han, Dae-Soo Kim, Mi-Young Son, Hyun-Soo Cho

**Affiliations:** 1 Korea Research Institute of Bioscience and Biotechnology, Daejeon, 34141 Republic of Korea; 2 Korea University of Science and Technology, Daejeon, 34316 Republic of Korea; 3 Department of Biological Science, Sungkyunkwan University, Suwon, 16419 Republic of Korea

**Keywords:** Oncogenes, Gene silencing

## Abstract

Epigenetic modifiers (miRNAs, histone methyltransferases (HMTs)/demethylases, and DNA methyltransferases/demethylases) are associated with cancer proliferation, metastasis, angiogenesis, and drug resistance. Among these modifiers, HMTs are frequently overexpressed in various cancers, and recent studies have increasingly identified these proteins as potential therapeutic targets. In this review, we discuss members of the SET and MYND domain-containing protein (SMYD) family that are topics of extensive research on the histone methylation and nonhistone methylation of cancer-related genes. Various members of the SMYD family play significant roles in cancer proliferation, metastasis, and drug resistance by regulating cancer-specific histone methylation and nonhistone methylation. Thus, the development of specific inhibitors that target SMYD family members may lead to the development of cancer treatments, and combination therapy with various anticancer therapeutic agents may increase treatment efficacy.

## SMYD proteins: potential therapeutic targets for cancer treatment

Understanding cancer growth and spread is vital for creating new treatments. Histone methyltransferases play a key role in cancer by controlling genes that can either encourage or inhibit tumor growth. This review focuses on the SMYD family of HMTs, which are associated with cancer progression, spread, and resistance to chemotherapy. The researchers studied the structure, function, and effects of different SMYD family members on cancer, using clinical data and biological experiments. The review also explores how these enzymes can be targeted by specific inhibitors, potentially offering new cancer treatments. The findings suggest that targeting SMYD family members, especially SMYD2 and SMYD3, could be an effective cancer treatment strategy. By developing drugs that specifically inhibit SMYD2 and SMYD3, researchers hope to provide new, more effective treatments for cancer patients. This summary was initially drafted using artificial intelligence, then revised and fact-checked by the author.

## Introduction

Epigenetic processes play crucial roles in various stages of cancer growth. DNA methylation, histone modification (including methylation, acetylation, phosphorylation, and ubiquitylation), and microRNAs (miRNAs) are associated with the regulation of oncogene and tumor suppressor gene expression^1–4^. Recently, through public databases such as The Cancer Genome Atlas (TCGA) database, it has been observed that the expression levels of epigenetic modifiers are up- or downregulated in cancer cells, suggesting their involvement in cancer-related processes^5–7^.

Among epigenetic modifiers, histone methyltransferases (HMTs), which are responsible for histone methylation in cancer-related epigenetic processes, are involved in regulating the expression of various oncogenes and tumor suppressor genes by altering euchromatin and heterochromatin structures^8^. Consequently, the expression of genes related to cancer growth, metastasis, and chemotherapy resistance is regulated by HMTs, making these enzymes potential therapeutic targets for cancer treatment^9^. In recent developments in cancer therapy, specific inhibitors, such as tazemetostat, which targets enhancer of zeste homolog 2 (EZH2) and thereby mediates histone H3 lysine (K) 27 methylation, have been approved by the Food and Drug Administration (FDA) for the treatment of follicular lymphoma and are currently in use. Moreover, various clinical studies are being conducted to explore the possibility of applying these inhibitors to treat different cancers^10^. The successful development of EZH2 inhibitors has led to active research on the mechanisms by which various HMTs function, and these studies have shown that HMTs have potential for use as crucial targets for cancer treatment.

Therefore, the aim of this review was to describe the SET and MYND domain-containing protein (SMYD) family, which has recently been reported to be associated with cancer growth, metastasis, and chemotherapy resistance. This review also aims to determine the potential of SMYD family members as therapeutic targets for cancer treatment.

## SMYD family members

**Fig. 1:**
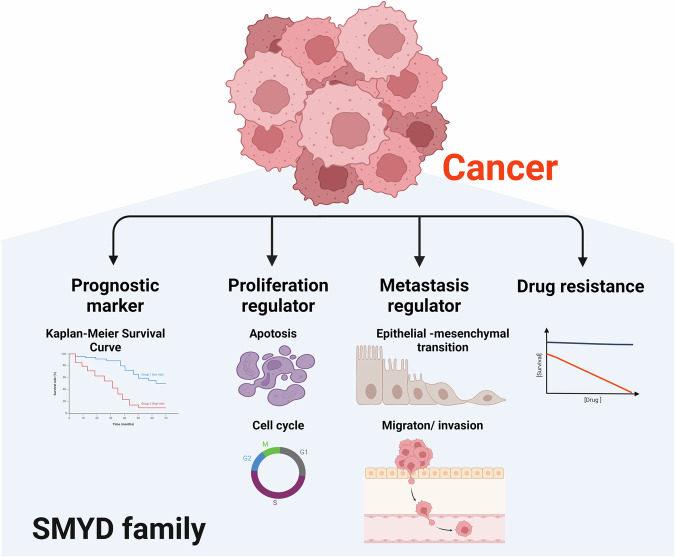
Functions of the SMYD family in cancer. The SMYD family is overexpressed in cancer and functions as a prognostic marker, proliferation regulator, and metastasis regulator. The SMYD family also plays a role in regulating drug resistance.

The SMYD family includes SMYD1, SMYD2, SMYD3, SMYD4, and SMYD5, which are responsible for the monomethylation of histone H3 lysine (K) 4 and the dimethylation of H3K36^11^. The SMYD family shares a similar structure. All members of the SMYD family commonly possess the SET (suppressor of variegation, enhancer of zeste, trithorax) and MYND (myeloid-nervy-DEAF1) domains, which are involved in protein interactions. Additionally, SMYD family members have a cysteine-rich post-SET domain. Unlike other members of the SMYD family, SMYD4 possesses a TPR (tetratricopeptide) domain^11,12^. Through the modification of these active markers, SMYD proteins play crucial roles in regulating the expression of various oncogenes, thus contributing to cancer growth and metastasis. Additionally, analysis of diverse clinical data has revealed that the expression levels of SMYD family members can be used as markers to predict poor prognosis^13^. Furthermore, the methylation of nonhistone proteins, such as p53, by SMYD family members is an important type of cancer-specific posttranslational modification (PTM) that is mediated by SMYD family members. Currently, most research on SMYD family members has focused on SMYD2 and SMYD3. Although SMYD4 and SMYD5 have recently been associated with cancer, few studies have investigated these associations (Fig. 1).

## SMYD1

Extensive studies on the functional role of SMYD1 in cancer have not been conducted, but research on its role as a prognostic marker of various cancer types is ongoing. According to RNA-seq data analysis, SMYD1 expression is suppressed in gastric cancer, but a negative correlation between SMYD1 expression and overall and progression-free survival has been reported^13^. Additionally, in gastric cancer, high expression of ankyrin repeat and SOCS box-containing 5 (ASB5), secreted frizzled-related protein 1 (SFRP1), SMYD1, and tachykinin receptor 2 (TACR2) is associated with short survival times and high risk in these patients^14^. Conversely, in breast cancer, SMYD1 expression is lower than that of other SMYD family members, and Kaplan‒Meier curve analysis indicates that lower SMYD1 expression is correlated with poor prognosis^15^. Moreover, transcriptome analysis of prostate cancer has revealed increased expression of various HMTs. Among these HMTs, EZH2, SETD5, SMYD1, and SUV420H2 are utilized in prognostic panels^16^. Therefore, these findings suggest that SMYD1 could serve as a potential prognostic marker in various cancers.

**Fig. 2:**
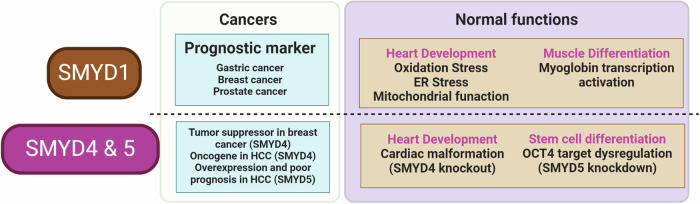
Overview of the functions of SMYD1, SMYD4, and SMYD5. In cancer, SMYD1, SMYD4, and SMYD5 serve as prognostic markers. The normal functions of SMYD1, SMYD4, and SMYD5 include regulating heart development, muscle differentiation, and stem cell differentiation.

Various functions of SMYD1 have been reported in normal cells. In the developing heart, immune-affinity tandem mass spectrometry (IP-MS/MS) confirmed that CDH4 is directly bound to SMYD1, suggesting that SMYD1 is necessary for heart development through the regulation of cadherin 4 (CDH4). Additionally, genomic analyses (RNA-seq, ATAT-seq) have indicated that SMYD1 is involved in the responses to angiogenesis, glycolysis, and hypoxia^17^. Furthermore, Smyd1 upregulation in mouse heart development and D-galactose-treated H9C2 cells suggested that Smyd1 plays a crucial role in regulating oxidative stress and ER stress in aging cardiomyocytes^18,19^. Smyd1 overexpression also suggests that Smyd1 is involved in cardiac differentiation and functional maintenance by suppressing Msi2-induced cardiac malfunction and mitochondrial dysfunction^20^. SMYD1 plays important roles not only in heart function but also in muscle function. Dynamic transcriptome profile analysis using Shaziling pigs revealed a potential association between SMYD1 and muscle growth^21^. Additionally, SMYD1 was confirmed to regulate myoglobin transcriptional activation through lysine 1975 methylation of the skeletal muscle-specific splice variant of the nascent polypeptide-associated complex (skNAC), suggesting that SMYD1 methylation is involved in muscle function^22^. In a study on smoking-induced skeletal muscle dysfunction, the abnormal differentiation of C2C12 cells induced by 5% cigarette smoke extract (CSE) involved the suppression of the Smyd1-H3K4me2-Purinergic Receptor P2X 7 (P2RX7) axis^23^. Therefore, SMYD1 plays a crucial role in muscle differentiation and function (Fig. 2).

## SMYD2

**Fig. 3:**
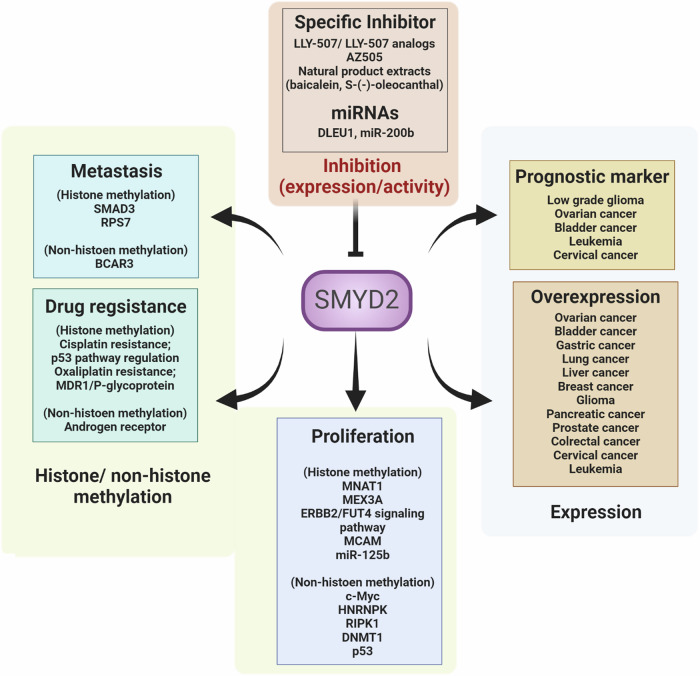
Overview of the cancer-related functions of SMYD2 and methods of SMYD2 inhibition (inhibition of its expression or activity) in cancers. SMYD2 is overexpressed and regulates cancer proliferation, metastasis, and drug resistance through histone methylation and nonhistone methylation. SMYD2 can also be used as a prognostic marker.

SMYD2 plays a role in upregulating gene expression via H3K4 monomethylation and H3K36 mono- and dimethylation^24^. This conclusion is supported by an increasing number of reports on the regulation of cancer-related genes that are associated with the proliferation and metastasis of various cancers. Moreover, in terms of cellular localization, SMYD2 is predominantly localized to the cytoplasm, unlike other HMTs, indicating a role for this protein not only in histone methylation but also in the methylation of cancer-related nonhistone proteins^25^. In addition, reports on SMYD2 inhibitors have highlighted the potential of SMYD2 as an important therapeutic target in various cancers. In this section, we describe how SMYD2 overexpression, which is observed in various cancers, influences proliferation, metastasis, drug resistance, and other processes through the regulation of cancer targets (Fig. 3 and Table 1).

**Table 1:** SMYD2 functions in cancer.

Functions	Description	References
Proliferation	SMYD2-mediated MNAT1 upregulation was suggested to activate the PI3K/AKT pathway in pancreatic cancer	^27^
	SMYD2 knockdown suppresses proliferation via the positive regulation of c-Myc expression in prostate cancer	^28^
	SMYD2 regulates H3K36me2 levels on MEX3A in colorectal cancer	^28^
	SMYD2 regulates the ERBB2/FUT4 signaling pathway in colorectal cancer	^29^
	SMYD2 upregulates the MCAM gene and is involved in regulating cancer stem cell characteristics in breast cancer	^33^
	SMYD2 knockdown increases apoptotic cell death and reduces anchorage-independent transformation in leukemia	^35^
	SMYD2 overexpression regulates miR-125b expression in renal cancer	^38^
	SMYD2 increases RNA-binding activity via the monomethylation of K422 on HNRNPK in colorectal cancer	^39^
	SMYD2 binding to RIPK1 inhibits RIPK1 phosphorylation in colorectal cancer	^40^
	SMYD2 positively regulates DNMT1 in colorectal cancer	^42^
	SMYD2 regulates the expression of glucose metabolism-related genes by changing the methylation of p53 at K370 in cervical cancer	^43^
Metastasis	Monomethylation of BCAR3 lysine K334 by SMYD2 regulates the actin cytoskeleton	^44^
	SMYD2 directly controls SMAD3 expression in lung cancer	^45^
	SMYD2 regulates RPS7 expression in lung cancer	^46^
Drug resistance	SMYD2 regulates AR protein stability through nonhistone methylation in prostate cancer	^57^.
	SMYD2 inhibition increases cell apoptosis via regulation of the p53 pathway (p21, Bax, and GADD45) in lung cancer	^58^
	Oxaliplatin sensitivity is controlled via regulation of MDR1/P-glycoprotein by SMYD2 in colorectal cancer	^59^
SMYD2 inhibitors and miRNAs	LLY-507 inhibits lung cancer tumor growth	^47^
	LLY-507 increases the levels of Sub-G1 and cleaved PARP	^49^
	OC reduces SMYD2 expression	^50^
	Baicalein inhibits SMYD2 expression	^51^
	The long non-coding RNA DLEU1 binds to SMYD2 and translocates to the APOC1 gene promoter	^47^
	Negative correlation between miR-200b expression and SMYD2 expression	^52^

*MNAT1* MNAT1 component of CDK-activating kinase, *MEX3A* Mex-3 RNA-binding family member A, *ERBB2* Erb-B2 receptor tyrosine kinase 2, *FUT4* fucosyltransferase 4, *MCAM* melanoma cell adhesion molecule, *RIPK1* receptor-interacting serine/threonine kinase 1, *DNMT1* DNA methyltransferase 1, *BCAR3* BCAR3 adapter protein, NSP family member, *SMAD3* SMAD family member 3, *RPS7* ribosomal protein S7, *OC* S-(-)-oleocanthal, *DLEU1* deleted in lymphocytic leukemia 1, *APOC1* apolipoprotein C1.

## SMYD2 in cancer proliferation

Recent reports have indicated increased SMYD2 expression in various cancers. Yadav et al. analyzed TCGA data and reported SMYD2 overexpression in various cancers. SMYD2 overexpression, which is correlated with overall survival (OS) and disease-free survival (DFS), suggests that SMYD2 could be used as a biomarker of various cancers^26^. In pancreatic cancer, SMYD2 was found to be overexpressed, and SMYD2 upregulated the MNAT1 component of CDK-activating kinase (MNAT1), which is a component of cyclin-dependent kinase (CDK)-activating kinase, via H3K36me2 modification in the promoter region. This SMYD2-mediated MNAT1 upregulation activates the phosphoinositide 3-kinase (PI3K)/AKT serine/threonine kinase (AKT) pathway, leading to increased proliferation of pancreatic cancer cells^27^. Similarly, SMYD2 is overexpressed in prostate cancer, and high SMYD2 expression is associated with poor prognosis. SMYD2 knockdown in prostate cancer cell lines suppresses proliferation via positive regulation of c-Myc expression^28^. Moreover, SMYD2 overexpression has been confirmed in colorectal cancer tissues from patients, and SMYD2 knockdown reduced cell proliferation in xenograft and in vitro models. In terms of direct targets, SMYD2 regulates the level of H3K36me2 on Mex-3 RNA-binding family member A (MEX3A), thereby controlling the growth of colorectal cancer by regulating the target of MEX3A, namely, caudal type homeobox 2 (CDX2). Furthermore, SMYD2-mediated regulation of the Erb-B2 receptor tyrosine kinase 2 (ERBB2)/fucosyltransferase 4 (FUT4) signaling pathway controls colorectal cancer growth^29,30^. In addition, a computational approach revealed a high rate of genetic alteration in SMYD2 in 360 hepatocellular carcinoma patient samples^31^. SMYD2 overexpression has been confirmed in ovarian cancer patient tissues, and SMYD2 knockdown inhibited the proliferation of ovarian cancer cell lines^32^. In breast cancer, the increase in H3K36me2 by SMYD2 is involved in upregulating the melanoma cell adhesion molecule (MCAM) gene and regulating the characteristics of cancer stem cells^33^. Moreover, treatment with the SMYD2 inhibitor AZ505 increased the sensitivity of glioma cells to cisplatin and similar chemotherapeutic agents^34^. Additionally, SMYD2 overexpression has been observed in leukemia. Knockdown of SMYD2 increases apoptotic cell death and reduces anchorage-independent transformation^35^. Furthermore, in pediatric B lineage acute lymphoblastic leukemia patients, SMYD2 expression is associated with poor prognosis^36^. In cervical cancer, SMYD2 expression is correlated with the Federation of Gynecology and Obstetrics (FIGO) stage, tumor size, and poor prognosis. SMYD2 knockdown via shRNA similarly suppresses the growth of cervical cancer cell lines^37^. In renal cancer, SMYD2 overexpression regulates miR-125b expression. The upregulation of miR-125b subsequently regulates renal cancer cell proliferation, invasion, and migration^38^.

Furthermore, SMYD2 reportedly plays an important role in the growth of various cancers through nonhistone methylation. In hepatocellular carcinoma, SMYD2 overexpression increases proliferation by directly binding to c-Myc and increasing its protein stability^39^. In colorectal cancer, SMYD2 increases RNA-binding activity via the monomethylation of K422 on HNRNPK, thereby influencing the stability of EGF-like domain multiple 7 (EGFL7) mRNA. Additionally, the binding of SMYD2 to receptor-interacting serine/threonine kinase 1 (RIPK1) inhibits RIPK1 phosphorylation, leading to the suppression of apoptosis and necroptosis^40,41^. Moreover, the positive regulation of DNA methyltransferase 1 (DNMT1) by SMYD2, through its binding, increases methylation in the promoter region of APC regulator of WNT signaling pathway 2 (APC2), resulting in negative regulation and increased growth of colorectal cancer^42^. In cervical cancer, SMYD2 regulates the expression of glucose metabolism-related genes by altering the methylation of p53 at K370^43^.

Therefore, SMYD2 overexpression is a significant factor in the growth of various cancers. Its role in positively regulating the expression of genes that are necessary for cancer growth supports its importance in cancer cell proliferation. Additionally, the identification of nonhistone methylation of various cancer-related genes suggests that SMYD2 is an important therapeutic target for cancer treatment.

## SMYD2 as a regulator of cancer metastasis

SMYD2 plays a significant role in metastasis by regulating the expression of various cancer-related genes. SMYD2 regulates breast cancer metastasis by controlling cytoskeleton remodeling. Specifically, through the monomethylation of lysine K334 in the adapter protein NSP family member (BCAR3), SMYD2 induces the recognition of the methyl-binding domain in formin-like (FMNL) proteins, thereby regulating the actin cytoskeleton. Through this action, it has been confirmed lamellipodia formation, an important feature of cell movement, is regulated by SMYD2 methylation^44^. Furthermore, SMYD2 regulates lung cancer metastasis by directly controlling SMAD family member 3 (SMAD3), which regulates the progression of lung cancer. The establishment of an in vitro epithelial–mesenchymal transition (EMT) system confirmed that the migration and invasion of highly invasive lung cancer cell lines are regulated by SMYD-SMAD3^45^. Additionally, the downregulation of SMYD2 controls the metastasis of lung adenocarcinoma through the regulation of ribosomal protein S7 (RPS7) expression^46^.

Therefore, SMYD2 plays a crucial role in inducing changes in the epithelial–mesenchymal transition (EMT) and remodeling of the cytoskeleton in cancer metastasis. Inhibiting SMYD2 with SMYD2-specific inhibitors effectively suppresses metastasis, which is the most problematic aspect of cancer treatment, thus increasing the success of cancer treatment.

## Regulation of SMYD2 with SMYD2-specific inhibitors and miRNAs

As described above, the regulation of SMYD2 expression controls the growth and metastasis of cancer, indicating that SMYD2 is a therapeutic target for cancer treatment. Various inhibitors, including natural compounds, are being developed to regulate SMYD2 expression/activity. Treatment with LLY-507, a specific inhibitor of SMYD2, has been shown to inhibit lung cancer tumor growth^47^. Furthermore, novel analogs of LLY-507 have been developed via chemical modification, and these analogs inhibit the growth of the gastric cancer cell lines AGS and NCI-87 more effectively than does LLY-507^48^. In ovarian cancer cell lines, increased sub-G1 and cleaved poly(ADP‒ribose) polymerase (PARP) levels were observed after treatment with SMYD2 siRNA and LLY-507, and a synergistic effect with PARP inhibitors was observed, confirming the utility of SMYD2 inhibitors in ovarian cancer^49^. In prostate cancer, treatment with S-(-)-oleocanthal (OC), which is a component of a Mediterranean extra virgin olive oil-rich diet, leads to decreased SMYD2 expression and regulation of the mechanistic target of rapamycin kinase (mTOR), mitogen-activated protein kinase (MAPK), and p65 signaling pathways, inhibiting prostate cancer growth both in vitro and in vivo^50^. Additionally, treatment with baicalein, which is a flavonoid from *Scutellaria baicalensis*, inhibits SMYD2 expression, suppressing lung cancer growth, migration, and invasion^51^.

Furthermore, miRNAs have recently been shown to regulate SMYD2 expression and activity. In gastric cancer, long non-coding RNA deleted in lymphocytic leukemia 1 (DLEU1) binds to SMYD2 and translocates to the apolipoprotein C1 (APOC1) gene promoter. The subsequent H3K4me3 modification leads to increased APOC1 expression, resulting in increased cell proliferation and glucose uptake in gastric cancer^47^. Conversely, miR-200b is expressed at low levels in hepatocellular carcinoma. A negative correlation has been noted between miR-200b and SMYD2 expression, and the overexpression of miR-200b suppressed SMYD2 expression. This inhibition of SMYD2 expression suppresses the proliferation of MHCC-91L hepatocellular carcinoma cells by inhibiting cyclin E1 and increasing P53 expression^52^. Therefore, the development of SMYD2 inhibitors is expected to be a crucial step in the development of cancer therapeutics, and the development of other approaches to regulate SMYD2, such as miRNAs, that can be combined with these inhibitors is anticipated to result in synergistic effects in important cancer treatments.

## SMYD2 as a prognostic marker

Previous reports have described SMYD2 expression and its inclusion in prognostic signatures for cancer diagnosis. Yu et al. analyzed transcriptome data from 711 low-grade gliomas (LGGs) and demonstrated that an epigenetic signature of 13 genes could strongly predict poor overall survival in patients with LGGs. Among these genes, the inhibition of SMYD2 by treatment with LLY-507 and shRNA suppressed glioma cell growth, indicating the significant oncogenic role of SMYD2^53^. Additionally, using RNA-seq data analysis of early-stage (I and II) ovarian carcinoma patients, 29 histotype-specific biomarkers, including SMYD2, were discovered. Analysis of the expression levels of these biomarkers could facilitate decision-making related to the clinical treatment of ovarian carcinoma patients^54^. The association of SMYD2 with overall survival was confirmed by analyzing differentially expressed genes (DEGs) between bladder cancer tissues and normal tissues^55^. Therefore, SMYD2 expression could be utilized as a prognostic marker for various cancer types.

## SMYD2 in drug resistance

Drug resistance has become a significant issue in cancer treatment, and recently, various epigenetic modifiers have been reported to be associated with drug resistance^56^. Recent studies have also investigated the crucial role of SMYD2 in inhibiting resistance to chemotherapy in various cancers. In prostate cancer, the androgen receptor (AR) plays a vital role in the growth of prostate cancer and castration-resistant prostate cancer. SMYD2 overexpression in prostate cancer regulates AR protein stability through the nonhistone methylation of this protein. This regulation leads to resistance to enzalutamide, and treatment with AZ505 has been confirmed to inhibit enzalutamide resistance^57^. Additionally, the inhibition of SMYD2 in non-small cell lung cancer increases sensitivity to cisplatin. This effect was supported by the fact that SMYD2 inhibition increased cell apoptosis via regulation of the p53 pathway (p21, Bax, and GADD45)^58^. SMYD2 knockdown in colon cancer increases the sensitivity of oxaliplatin-resistant cells to oxaliplatin treatment. These findings confirmed that oxaliplatin sensitivity is controlled via the regulation of MDR1/P-glycoprotein by SMYD2^59^. Thus, SMYD2 plays an important role in inhibiting resistance to cancer chemotherapy, and simultaneous treatment with SMYD2 inhibitors during chemotherapy is expected to increase the effectiveness of chemotherapy.

## Other functions of SMYD2

SMYD2 is overexpressed in various cancers and is associated with their functions. However, SMYD2 is also related to the functions of normal cells and other diseases. In bone formation, the methylation of EZH2 regulated by SMYD2 controls osteoblast differentiation^60,61^. In an angiotensin II (Ang II)-induced vascular endothelial cell (VEC) senescence model, SMYD2 was shown to be upregulated, regulating the expression of the aging-related genes cyclin-dependent kinase inhibitor 1A (Cdkn1a) and Cdkn2a, suggesting that SMYD2 is a potential therapeutic target for vascular aging^62^. Additionally, during blood‒brain barrier (BBB) disruption, Smyd2 expression is upregulated. In peri-ischemic brains, upregulated Smyd2 induces sphingosine kinase (Sphk)/sphingosine-1-phosphate receptor (S1PR) methylation, affecting protein stability and damaging endothelial integrity, indicating that the functions of SMYD2 are related to vascular structure^63^. In terms of the association between the immune response and SMYD2, the phosphorylation of interferon regulatory factor 3 (IRF3), a key component of the antiviral innate response, is regulated by SMYD2. In macrophages, SMYD2 binds to IRF3, promoting the binding of the phosphatase PP1alpha and inhibiting IRF3 phosphorylation. This affects the production of type 1 interferons in response to virus infection, thereby reducing the antiviral innate response regulated by SMYD2^64^. Additionally, SMYD2 methylation of TNF receptor-associated factor 2 (TRAF2) leads to increased nuclear factor kappa B (NF-κB) signal transduction, suggesting that SMYD2 can regulate both acute and chronic inflammation^65^. In the kidney, SMYD2 is a key factor in the induction of renal fibrosis. In a murine model of renal fibrosis, SMYD2 is overexpressed, which induces fibrosis by inhibiting the phosphorylation of profibrotic signaling molecules such as Smad3 and AKT signaling. These findings indicate that SMYD2 could be an important target in kidney disease^66,67^.

## SMYD3

**Fig. 4:**
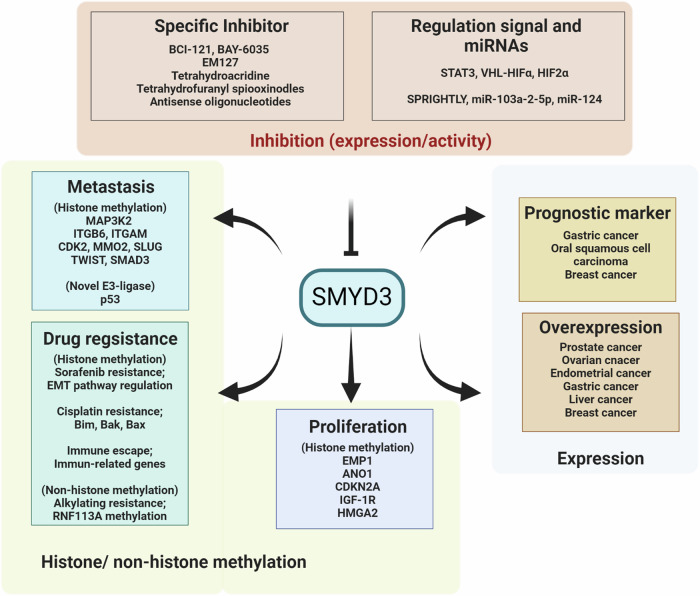
Roles of SMYD3 in cancer and strategies for inhibiting SMYD3 expression and activity during oncogenesis. SMYD3 is overexpressed and regulates cancer proliferation, metastasis, prognosis, and drug resistance.

SMYD3, which is a member of the SMYD lysine methylase family, plays a critical role in the methylation of many histone and nonhistone proteins. Functionally, SMYD3 facilitates H3K4 modification, catalyzing lysine methylation to regulate downstream gene transcription^68^. Moreover, SMYD3 can bind to specific DNA motifs within promoter regions of target genes and interact with RNA polymerase II^68^. Aberrant SMYD3 expression is associated with carcinogenesis, and evidence indicates that it is involved in promoting the expression of critical oncogenes in cancer, including breast, colorectal, gastric, liver, and ovarian cancers^68–70^. In this section, we describe SMYD3-mediated regulatory mechanisms that are implicated in the progression of several cancers, including mechanisms that regulate cancer cell proliferation, metastasis, and drug resistance. Additionally, we address the potential of SMYD3 inhibitors as promising strategies for cancer treatment (Fig. 4 and Table 2).

**Table 2:** SMYD3 functions in cancer.

Functions	Description	References
Proliferation	SMYD3 depletion in endometrial cancer reduces cell proliferation	^71^
	SMYD3 expression is increased in gastric cancer and promotes proliferation by epigenetically reducing EMP1	^72^
	Transcriptionally represses target gene by associating with the NuRD complex in HCC	^73^
	Increases ANO1 expression and regulates cancer cell proliferation in NSCLC	^74^
	SMYD3 enhances NSCLC and CRC cell proliferation	^75,76^
	Silencing of SMYD3 upregulates S-phase arrest genes and increases cell proliferation in ovarian cancer	^77^
	SMYD3 activates AKT signaling by directly regulating IGF-1R, inducing cell proliferation in breast cancer	^78^
	SMYD3 enhance cancer cell stemness and proliferation by increasing HMGA2 expression	^79^
Metastasis	SMYD3-dependent methylation of MAP3K2 promotes EMT by regulating vimentin in prostate cancer	^80^
	SMYD3 KD inhibits tumor metastasis by regulating ITGB6 and ITGAM in ovarian cancer	^81^
	Overexpression in epithelial ovarian cancer promotes metastasis by decreasing p53 protein stability	^82^
	Overexpression in HCC promotes tumorigenicity and intrahepatic metastasis by upregulating CDK2 and MMP2 expression	^83^
	ANKHD1 and SMYD3 coexpression is associated with poor prognosis in HCC	^84^
	Promotes EMT by interacting with SMAD3 in breast cancer	^86^
	Positive feedback loop of TGFβ1-SMYD3-ITGB6 promotes invasion and adhesion in ovarian cancer	^87^
Drug resistance	SMYD3 is involved in SMAD2/3-mediated EMT in sorafenib-resistant HCC cells	^88^
	SMYD3 overexpression increases cisplatin resistance	^75,89,90^
	Major regulator of sensitivity to alkylation-based chemotherapy in squamous cell carcinoma	^91^
	Smyd3 depletion induces the infiltration of CD8+ T cells and increases sensitivity to anti-PD-1 therapy in HPV-negative head and neck squamous cell carcinoma	^92^
SMYD3 inhibitors	BAY-6035 specifically inhibits MAP3K2 methylation via SMYD3	^93^
	EM127 exhibits selectivity for Cys186 of SMYD3	^94^
	Compound 29 is a novel class of tetrahydroacridine	^95^
	Compound 7r exhibits potent inhibitory effect on SMYD3 with anticancer effects	^97^
	Smyd3-ASOs suppresses liver tumor growth in a mouse model of chemical-induced HCC	^98^

*EMP1* epithelial membrane protein 1, *HCC* hepatocellular carcinoma, *ANO1* anoctamin-1, *NSCLC* non-small cell lung cancer, *CRC* colorectal cancer, *IGF-1R* insulin-like growth factor 1 receptor, *HMGA2* high mobility group AT-hook 2, *MAP3K2* mitogen-activated protein kinase kinase kinase 2, *ITGB6* integrin subunit beta 6, *ITGAM* integrin subunit alpha M, *CDK2* cyclin-dependent kinase 2, *MMP2* matrix metallopeptidase 2, *ANKHD1* ankyrin repeat and KH domain containing 1, *SMAD3* SMAD family member 3, *Bak* BCL2 antagonist/killer, *Bax* BCL2-associated X, *ASO* antisense oligonucleotide.

## SMYD3 in cancer proliferation

Numerous previous studies have reported that SMYD3 is upregulated in several cancers; therefore, SMYD3 is closely associated with disease progression. SMYD3 promotes cell proliferation by regulating the expression of genes involved in the cell cycle, proliferation, and apoptosis.

Both in vitro and in vivo experiments have shown that SMYD3 depletion in endometrial cancer reduces cell proliferation and compromises nonhomologous end joining (NHEJ) repair. Moreover, the SMYD3 inhibitor BCI-121, which is utilized to treat endometrial cancer, attenuates the tumorigenicity of endometrial cancer and improves the efficacy of radiotherapy^71^. Moreover, SMYD3 expression is significantly elevated in gastric cancer tissues and is correlated with aggressive clinical features and poor prognosis. SMYD3 promotes gastric cancer cell proliferation by epigenetically reducing epithelial membrane protein 1 (*EMP1*) expression in an H4K20me3-dependent manner^72^. In hepatocellular carcinoma, SMYD3 transcriptionally represses target gene expression by associating with the NuRD (MTA1/2) complex to regulate proliferation^73^. In non-small cell lung cancer, SMYD3 binds to the anoctamin-1 (ANO1) promoter region, which is enriched with H3K4me3, thereby increasing ANO1 transcription and regulating cancer cell proliferation^74^. Additionally, SMYD3 overexpression promotes non-small cell lung cancer cell proliferation, whereas SMYD3 knockdown inhibits cell proliferation^75^. In colorectal cancer, SMYD3 affects proliferation in vitro, and silencing SMYD3 suppresses tumor growth in xenograft-bearing mice^76^. Similarly, Jiang et al. reported that silencing SMYD3 inhibits ovarian cancer cell proliferation in vitro by arresting cell cycle progression in the S phase. Moreover, SMYD3 knockdown upregulates the expression of the cyclin-dependent kinase (CDK) inhibitors CDKN2A (p16INK4), CDKN2B (p15INK4B), CDKN3, and cell division cycle 25A (CDC25A), which are related to S-phase arrest. Specifically, SMYD3 binds to the promoter region of CDKN2A, downregulating its expression level via H3K4me3^77^. In breast cancer, SMYD3 expression is significantly upregulated and correlated with shorter patient survival. SMYD3 depletion inhibits cell proliferation, colony formation, and xenograft tumor growth. Furthermore, SMYD3 directly regulates insulin-like growth factor 1 receptor (IGF-1R), which is a critical activator of AKT; thus, the SMYD3/insulin-like growth factor 1 receptor (IGF-1R)/AKT/E2F transcription factor 1 (E2F-1) axis forms a positive feedback loop that activates AKT signaling in breast cancer^78^. According to machine learning data from 429 chromatin regulators, SMYD3 is aberrantly expressed in oral squamous cell carcinoma, and SMYD3 expression is associated with oral squamous cell carcinoma formation and poor prognosis. Functional studies have demonstrated that SMYD3 enhances cancer cell stemness and proliferation in vitro, as well as tumor growth in vivo. Furthermore, SMYD3 binds to the high mobility group AT-hook 2 (HMGA2) promoter, increasing H3K4me3 levels and transactivating HMGA2^79^.

## SMYD3 in cancer metastasis

In prostate cancer, SMYD3 is frequently overexpressed and plays a critical role in regulating tumor-associated phenotypes through its methyltransferase activity. SMYD3-dependent methylation of mitogen-activated protein kinase kinase kinase 2 (MAP3K2) promotes EMT by regulating vimentin expression. Furthermore, activation of the SMYD3 and MAP3K2 signaling pathways results in the formation of a positive feedback loop that promotes SMYD3 expression, indicating its involvement in metastatic prostate cancer^80^. In ovarian cancer, shRNA-mediated knockdown of SMYD3 decreases spheroid invasion and adhesion by suppressing integrin subunit beta 6 (ITGB6) and integrin subunit alpha M (ITGAM). Specifically, increased SMYD3 and H3K4me3 binding at the ITGB6 and ITGAM promoters is noted in spheroids compared with monolayer cells. However, inhibition of SMYD3 expression decreases the binding of these proteins to these promoter regions. Moreover, SMYD3 knockdown inhibits tumor metastasis and reduces ascites volume in both peritoneal seeding and patient-derived xenograft (PDX) models^81^. Additionally, SMYD3 is frequently overexpressed in epithelial ovarian cancer and promotes metastasis by directly decreasing p53 protein stability and promoting p53 ubiquitination, indicating that SMYD3 may be a novel E3 ligase of p53^82^. SMYD3 regulates the transcription of the CDK2 and matrix metallopeptidase 2 (MMP2) genes by directly binding to their promoter regions and increasing H3K4me3 modification. SMYD3 overexpression promotes the tumorigenicity and intrahepatic metastasis of hepatocellular carcinoma cells by upregulating CDK2 and MMP2 expression^83^. Zhou et al. reported that SMYD3 promotes the migration and invasion of hepatocellular carcinoma cells. They identified ankyrin repeat and KH domain containing 1 (ANKHD1) as a coregulator of SMYD3, and the promigratory and proinvasive effects of SMYD3 were attenuated when ANKHD1 was downregulated by siRNA. Furthermore, SMYD3 binds and activates the Snail family transcriptional repressor 2 (SNAI2) promoter, leading to increased levels of H3K4me3, H3K9Ac, and H3K14Ac; thus, the coexpression of SMYD3 and ANKDH1 is associated with poor prognosis in hepatocellular carcinoma patients^84^.

Interestingly, Liu et al. reported that long non-coding RNA (lncRNA) LTSCCAT promotes tongue squamous cell carcinoma metastasis by functioning as a competitive endogenous RNA that regulates miR-103a-2-5p. This microRNA negatively regulates SMYD3 by binding to the SMYD3 3′-UTR. SMYD3 directly regulates twist family BHLH transcription factor 1 (TWIST1) transcription via H3K4me3 modification. High expression of lncRNA LTSCCAT in TSCC promotes invasion and metastasis by increasing the level of the SMYD3/TWIST1 transcript by negatively regulating miR-103a-2-5p^85^. In addition, SMYD3 promotes EMT in breast cancer by directly interacting with SMAD3, but this interaction is not necessary for SMAD2/3 phosphorylation. SMYD3 knockdown or treatment with its inhibitor, BCI-121, reduces the transforming growth factor beta (TGFβ)-induced association of SMAD3 with chromatin. Treatment of mesenchymal-like MDA-MB-231 cells with BCI-121 has been shown to decrease the expression of mesenchymal genes and reduce invasion in a zebrafish xenograft model. Furthermore, higher SMYD3 levels are correlated with reduced metastasis-free survival in breast cancer patients^86^. Similarly, Jiang et al. reported that SMDY3 and ITGB6 activate the TGFβ1/Smad3 pathway, leading to the upregulation of Snail, vimentin and N-cadherin in 3D-cultured ovarian cancer spheroids. Moreover, TGFβ1 promotes SMYD3 and ITGB6 expression via a positive feedback loop, suggesting that activation of the SMYD3/ITGB6/TGFβ1/Smad3 axis promotes the invasion and adhesion of ovarian cancer spheroids^87^.

## SMYD3 in drug resistance

Abnormal SMYD3 expression has been reported in various cancers, and SMYD3 upregulation is associated with drug resistance. In a previous report, SMYD3 was markedly upregulated in sorafenib-resistant tumors and cells. Loss- and gain-of-function studies have shown that SMYD3 contributes to the migration, invasion, metastasis, and stemness of sorafenib-resistant hepatocellular carcinoma cells. Mechanistically, SMYD3 is required for SMAD2/3-mediated EMT in sorafenib-resistant cells, and it contributes to this process by interacting with SMAD2/3 and upregulating the expression of the SRY-box transcription factor 4 (SOX4), zinc finger E-box binding homeobox 1 (ZEB1), Snail family transcriptional repressor 1 (SNAIL1), and MMP9 genes^88^. In lung cancer, SMYD3 knockdown increases sensitivity, whereas SMYD3-overexpressing non-small cell lung cancer cells are more resistant to cisplatin-induced apoptosis because of the regulation of the Bim, BCL2 antagonist/killer (Bak), and BCL2-associated X (Bax) genes^75^. Lv et al. also reported that SMYD3 expression is correlated with clinicopathological stage and chemoresistance to cisplatin in non-small cell lung cancer. Moreover, high SMYD3 expression renders non-small cell lung cancer cells chemoresistant to cisplatin, and the mechanism is mediated by its coregulator ANKHD1^89^. In breast cancer, SMYD3 is related to chemoresistance to cisplatin. RNAi-mediated knockdown of SMYD3 increases sensitivity to cisplatin, whereas SMYD3 overexpression decreases sensitivity to cisplatin via the regulation of apoptosis. Researchers have reported decreased SMYD3 expression during cisplatin treatment, and its regulation may be related to microRNA-124 because the SMYD3 3’-UTR contains a miR-124 binding site^90^.

Other researchers have identified SMYD3 as a major regulator of sensitivity to alkylation-based chemotherapy in squamous cell carcinoma. They reported that SMYD3-mediated ring finger protein 113A (RNF113A) methylation impairs its interaction with the phosphatase PP4, thereby controlling RNF113A phosphorylation. This cross-talk between posttranslational modifications serves as a key switch in promoting and maintaining 113A (RNF113A) E3 ligase activity, which is essential for its role in the alkylation damage response. Consequently, SMYD3 inhibition increases sensitivity to alkylating chemotherapy^91^. In addition, SMYD3 has been shown to mediate immune escape in human papillomavirus (HPV)-negative head and neck squamous cell carcinoma. Nigam et al. reported that SMYD3 binds to the ubiquitin-like with PHD and ring finger domains 1 (UHRF1) transcript, which subsequently binds to H3K9me-enriched promoters of immune-related genes. Furthermore, SMYD3 represses immune-related genes through H4k20me3. In addition, Smyd3 depletion induces the infiltration of CD8+ T cells and increases sensitivity to anti-programmed cell death protein 1 (PD-1) therapy. Thus, combining a reduction in SMYD3 with immune checkpoint blockade could overcome anti-PD-1 resistance in HPV-negative head and neck squamous cell carcinoma^92^.

## Regulation of SMYD3: SMYD3-specific inhibitors

To date, several structures of inhibitors that have been confirmed to target SMYD3 have been identified, and these inhibitors are candidates for cancer therapy. Several research groups have continuously developed novel inhibitors that target SMYD3. Gradl et al. performed a thermal shift assay (TSA)-based high-throughput screening (HTS) with 410,000 compounds and identified a series of benzodiazepine-based SMYD3 inhibitors. After optimization and validation with surface plasmon resonance (SPR) analysis and isothermal titration calorimetry (ITC), the researchers reported that BAY-6035 exhibited nanomolar potency and selectivity. Given that SMYD3 methylates and regulates several nonhistone proteins, including MAP3K2, they investigated the effect of BAY-6035 on MAP3K2 methylation and reported that BAY-6035 could specifically inhibit MAP3K2 methylation via SMYD3 with an IC_50_ < 100 nM^93^.

Parenti et al. developed EM127, which is a 4-aminopiperidine-based compound bearing a 2-chloroethanoyl group as a reactive warhead. This compound is selective for Cys186, which is located in the substrate/histone binding pocket of SMYD3, as confirmed by mass spectrometry. Functionally, at low micromolar concentrations, EM127 strongly inhibited and attenuated MDA-MB-231 breast cancer cell and HCT116 colorectal cancer cell proliferation. Mechanistically, EM127 strongly decreased CDK2, c-MET, N-cadherin, and fibronectin 1 mRNA levels by inhibiting SMYD3^94^.

Additionally, Huang et al. identified irreversible SMYD3 inhibitors, which are a novel class of tetrahydroacridine compounds that function through a covalent mechanism. The optimization of these inhibitors led to the discovery of 4-chloroquinolines. Among these inhibitors, Compound 29 significantly reduced SMYD3 protein levels in 3D-cultured HepG2 cells compared with the DMSO control^95^. Another research group employed Schrodinger® software to screen libraries of small molecules in silico, aiming to identify a lead small molecule candidate for SMYD3 inhibition. Five compounds that were predicted to have high binding affinities for the SMYD3 binding pocket were assessed in breast cancer cell lines. Among these inhibitors, inhibitor-4 induces cell cycle arrest and apoptosis in breast cancer cells^96^. Furthermore, a structure-based drug design strategy was utilized to identify SMYD3 inhibitors. For example, Zhu et al. designed a series of novel tetrahydrofuranyl spirooxindoles for use as SMYD3 inhibitors on the basis of their structure. Biochemical analysis demonstrated that Compound 7r exhibited potent inhibitory effects on SMYD3 and displayed anticancer activity against stomach adenocarcinoma both in vitro and in vivo. A mechanistic investigation revealed that Compound 7r suppressed Akt methylation and induced lethal levels of autophagy by inhibiting the Akt-mTOR pathway, thereby regulating autophagic cell death by targeting SMYD3^97^.

Recently, Kontaki et al. demonstrated that targeting SMYD3 with next-generation antisense oligonucleotides (Smyd3-ASOs) efficiently modulated Smyd3 mRNA levels in vivo. Treatment with Smyd3-ASOs suppressed liver tumor growth in a mouse model of chemical-induced hepatocellular carcinoma and inhibited the tumor growth rate, migration, and capacity by blocking cellular dedifferentiation and the expansion of hepatic cancer stem cells^98^.

## Signals upstream of SMYD3

Several previous studies have reported factors that regulate SMYD3 in various cancers. Lin et al. reported that SMDY3 and STAT3 expression is positively correlated with the progression of chronic lymphocytic leukemia. SMYD3 upregulation promotes proliferation and suppresses the expression of apoptosis-related genes. Chromatin immunoprecipitation (ChIP) and promoter assays have revealed that signal transducer and activator of transcription 3 (STAT3) directly regulates the SMYD3 promoter region. Furthermore, the STAT3 inhibitor WP1066 has been shown to downregulate SMYD3 transcription by inhibiting the binding of STAT3 to the SMYD3 promoter, suppressing chronic lymphocytic leukemia cell growth in vivo^99^. Similarly, SMYD3 is directly regulated by STAT3 in chronic lymphocytic leukemia. STAT3 knockdown suppresses cell proliferation and invasion, whereas SMYD3 overexpression promotes cell proliferation and invasion^100^.

Another study revealed SMYD3 as a mediator between the von Hippel‒Lindau/hypoxia-inducible factor α (VHL-HIFα) axis and epidermal growth factor receptor (EGFR) in renal cell carcinoma. Liu et al. reported high SMYD3 expression in RCC, with functions related to cell proliferation, colony formation, and in vivo tumor growth. Mechanistically, SMYD3 cooperates with SP1 to increase EGFR expression, activating its downstream signals. Furthermore, HIF-2α directly binds to the SMYD3 promoter, inducing SMYD3 expression. Thus, EGFR activation mediated by the VHL/HIF-2α/SMYD3 signaling cascade promotes renal cell carcinoma progression^101^.

In addition, Lee et al. identified the lncRNA SPRIGHTLY gene as a regulator of SMYD3 pre-mRNA transcription in medulloblastomas. SPRIGHTLY binds to the intronic region of SMYD3 pre-mRNA and interacts with the PTPB1 protein to regulate SMYD3 exon skipping to produce an aberrant protein. SPRIGHTLY-driven SMYD3 regulation increases the expression of EGFR signaling genes in G4 medulloblastoma cells^102^.

## Other functions of SMYD3

SMYD3, an epigenetic modifier, plays crucial roles in cancer development and progression, as well as in the regulation of various biological processes in normal cells. In this section, we introduce the key functions of SMYD3 in normal cells. Codato et al. reported that SMYD3 overexpression in myoblasts promotes muscle differentiation and myoblast fusion, whereas silencing SMYD3 or pharmacological inhibitor treatment decreases muscle differentiation. Transcriptome analysis of murine myoblasts via the regulation of SMYD3 expression revealed that SMYD3 contributes to skeletal muscle differentiation by targeting the critical muscle regulatory factor myogenin^103^. Recently, Sajic et al. reported that SMYD3 regulates adipocyte precursor proliferation at the early steps of differentiation^104^. Other researchers have shown that SMDY3 expression is downregulated during both osteogenic and chondrogenic differentiation in human bone marrow MSCs^105^. Furthermore, Fittipaldi et al. demonstrated the key functions of SMYD3 under physiological conditions during development using mouse embryonic stem cells (ESCs) and zebrafish as model systems. SMYD3 depletion promotes the induction of mesodermal patterning during the in vitro differentiation of ESCs. Smyd3 knockdown in zebrafish induces the upregulation of mesendodermal markers during zebrafish gastrulation, indicating that SMYD3 modulates mesendodermal markers involved in development and ESC differentiation^106^.

## SMYD4, SMYD5, and other SMYD family members

The number of studies on SMYD4 and SMYD5 in the field of cancer has increased, although relatively few reports have compared SMYD4 and SMYD5 to SMYD2 and SMYD3. SMYD4 and SMYD5 are involved in cellular processes that are relevant to cancer development and progression, including gene regulation and cell signaling (Fig. 2).

The role of SMYD4 appears to be context dependent and can vary across different cancer types. In breast cancer, SMYD4 has been suggested to act as a tumor suppressor gene. Han et al. demonstrated that high expression of miR-1307-3p, which targets SMYD4, is associated with poor prognosis in breast cancer patients, indicating that miR-1307-3p potentially plays an oncogenic role by downregulating SMYD4 expression^107^. Conversely, in hepatocellular carcinoma, SMYD4 functions as an oncogene. Zhou et al. revealed that SMYD4 is highly upregulated in hepatocellular carcinoma and plays a role in promoting cell proliferation and metastasis. They identified protein arginine methyltransferase 5 (PRMT5) as an SMYD4-binding protein. Mechanistically, SMYD4 monomethylates PRMT5 and enhances the interaction between PRMT5 and MEP50, promoting the demethylation of H3R2 and H4R3 in the PRMT5 target gene promoter and resulting in the induction of disheveled segment polarity protein 3 (DVL3) expression and the inhibition of E-cadherin, RB transcriptional corepressor-like 2 (RBL2), and miR-29b-1-5p expression^108^. These findings highlight the complexity of the role of SMYD4 in cancer and emphasize the importance of considering its specific functions and mechanisms in the context of different cancer types. However, further research is needed to fully elucidate the molecular mechanisms underlying the dual role of SMYD4 in cancer progression.

A recent study by Chi et al. revealed the clinical significance of SMYD5 in hepatocellular carcinoma. These findings suggest that elevated SMYD5 expression is associated with cancer progression and poorer survival outcomes in patients with hepatocellular carcinoma. Furthermore, functional experiments revealed that SMYD5 downregulation reduces the proliferation, migration, and invasion of hepatocellular carcinoma cells. Interestingly, silencing SMYD5 also increases the sensitivity of hepatocellular carcinoma cells to paclitaxel, suggesting a potential role for SMYD5 in chemoresistance mechanisms^109^.

In the context of normal physiology, studies have reported that SMYD4 and SMYD5 are associated with development, stemness, and differentiation. Xiao et al. reported high smyd4 expression levels in the hearts of zebrafish. To determine the role of smyd4 in heart development, a smyd4 mutant zebrafish line (*smyd4*^L544Efs*1^) was generated using CRISPR/Cas9. They reported that maternal and zygotic *smyd4*^L544Efs*1^ mutants exhibited severe cardiac malformations, including defects in left–right patterning and looping and hypoplastic ventricles. Moreover, the researchers identified two rare SMDY4 genetic variants in a patient cohort with congenital heart defects, indicating that smyd4 functions as a key epigenetic regulator by altering histone modifications during cardiac development^110^. The role of SMYD5, a histone lysine methyltransferase, in stemness has been studied, and it has been reported that alterations in gene expression are associated with stem cell differentiation. Kidder et al. reported that depletion of SMYD5 leads to compromised self-renewal, including dysregulation of OCT4 target gene expression, resulting in altered differentiation. In this study, the authors suggested that SMYD5 plays a role in regulating embryonic stem cell maintenance by silencing lineage-specific genes^111,112^. However, research on the functions and mechanisms of SMYD4 and SMYD5, which have been relatively less studied, will help elucidate physiological phenomena in the human body in the future. Furthermore, SMYD4 and SMYD5 are expected to play important roles as key histone modifiers underlying the development of various diseases.

## Perspectives

In this review, we explored the cancer-related functions of various SMYD family members. The identification of targets that inhibit various characteristics of cancer is a crucial step in the development of cancer therapeutics. Recent research on SMYD family members has progressed rapidly in terms of understanding the roles of these proteins in cancer growth, metastasis, and resistance to anticancer agents. The research presented in this review indicates that SMYD family members are important targets for cancer therapy, with SMYD2 and SMYD3 proposed as more effective targets for cancer treatment. In particular, research and the development of specific inhibitors for SMYD2 and SMYD3 have demonstrated the potential of targeting these proteins to inhibit the growth and metastasis of various cancers. Additionally, the use of SMYD family-specific inhibitors has been suggested as a solution for the acquired resistance and low treatment efficacy that are observed during chemotherapy. Specifically, the administration of SMYD family-specific inhibitors during chemotherapy can increase the sensitivity of cancer cells to anticancer agents, leading to fewer side effects and more effective cancer treatment. However, although specific inhibitors of the SMYD family are currently being developed, there is a need for inhibitors that are highly specific for each member of the SMYD family because multiple functions of SMYD family members have been described in normal cells. Thus, inhibitors that selectively target cancer-specific processes related to growth, metastasis, and resistance to anticancer agents at low concentrations without affecting normal cells must be developed.
